# Internal Jugular and Subclavian Vein Thrombosis in a Post-liver Transplant Patient

**DOI:** 10.7759/cureus.6557

**Published:** 2020-01-03

**Authors:** Deema Algoblan, Luluwah AlAitah, Abdullah M Alotaibi

**Affiliations:** 1 Emergency Medicine, Alfaisal University, Riyadh, SAU; 2 Family Medicine, Alfaisal University, Riyadh, SAU; 3 Emergency Medicine, King Faisal Specialist Hosptial and Research Center, Riyadh, SAU

**Keywords:** internal jugular vein, venous thrombosis, upper extremity thrombosis, case report

## Abstract

Internal jugular vein thrombosis (IJVT) is an unusual case of vascular disease of the upper limb veins, that could result in multiple complications if left untreated. IJVT can be subdivided into primary and secondary. Primary IJVT is when the thrombosis happens to someone without known risk factors, while secondary IJVT is when it happens to a person with previous risk factors. Our patient is a 66-year-old male with a history of hypertension and is status post-liver transplant in 2014 due to end-stage liver disease; he presented to the emergency department of the King Faisal Specialist Hospital & Research Center complaining of progressive right chest, flank, and back pain for the past month. On physical examination, the patient had right upper limb, chest, and neck increase in vascular markings and right supraclavicular swelling with no erythema. Upper extremity and neck ultrasound showed positive deep vein thrombosis (DVT) of the right internal jugular vein, right subclavian vein, and axillary vein. A chest X-ray showed right-sided pleural effusion with no mediastinal shift. Computer tomography (CT) demonstrated thrombosed right internal jugular and subclavian veins. General internal medicine service was consulted and they started the patient on Emxparine 1 mg/kg twice daily. The patient improved and is doing fine. He is scheduled for repeated outpatient follow-ups.

## Introduction

Deep vein thrombosis (DVT) refers to the formation of one or more blood clots in the deep veins in the body. Most of the DVT happens in the lower leg or thigh, yet, it could still possibly occur in any deep vein within the body. A DVT can wreck and form an embolism that causes further serious complications in the location it travels to. The pathogenesis or mechanism of venous thromboembolism (VTE) was first described by Rudolf Virchow or the Virchow triad, which has three components as follows: vascular endothelial injury/dysfunction, hemodynamic changes (stasis, turbulence), and hypercoagulability [1-2].

Upper extremity deep venous thrombosis (UEDVT) involves thrombosis of the deep veins of the arm, as they finally reach the entrance of the thoracic cavity. Nowadays, UEDVT is growing in frequency and that is mostly due to the increased use of central venous catheters and cardiac devices. The internal jugular vein is most commonly affected; other veins that could also be affected are the subclavian, brachial, and basilic veins [3].

## Case presentation

A 66-year-old gentleman, known to have essential hypertension and is status post living related liver transplantation (right graft) in 2014 as therapy for end-stage liver disease secondary to cryptogenic cirrhosis vs autoimmune, presented to the emergency department at a tertiary care center with a one-month history of the progressive right chest, flank, and back pain. Pain is vague in character, mostly presenting as stinging and is intermittent in duration with no triggers or relievers. The patient tried taking over the counter painkillers with no noticeable relief. Pain is not positional and is not exacerbated by movement. There was no associated rash but the patient noticed increased vascular marking on his right upper extremity, chest, and neck. He denied any swelling or redness; however, he did notice a painless swelling in his right neck for two days. The patient reported persistent dry cough after pilgrimage (two months), and was resistant to treatment, with nonspecific pattern fever with no shortness of breath; he took a five-day course of antibiotics for the cough. There was no previous history of any excessive upper extremity exertion or recent catheterization in the neck. No personal or family history of thrombophilia or thromboembolic events were noted.

Vital signs on presentation were a blood pressure of 142/86 mmHg, heart rate of 76 bpm with oxygen saturation of 95% on room air, respiratory rate of 20 breaths/min and an oral temperature of 36.6 degrees C. On physical examination, patient came walking, alert, oriented in mild distress with no interference. The patient had right upper limb, chest, and neck increase in vascular markings with no erythema. There was a right supraclavicular swelling not associated with a palpable mass, erythema or any discoloration. Neurological assessment was unremarkable with good tone and 5 out 5 power bilaterally with normal active and passive range of motion bilaterally and intact sensations. Chest examination showed normal bilateral air entry with no added sounds.

**Figure 1:**
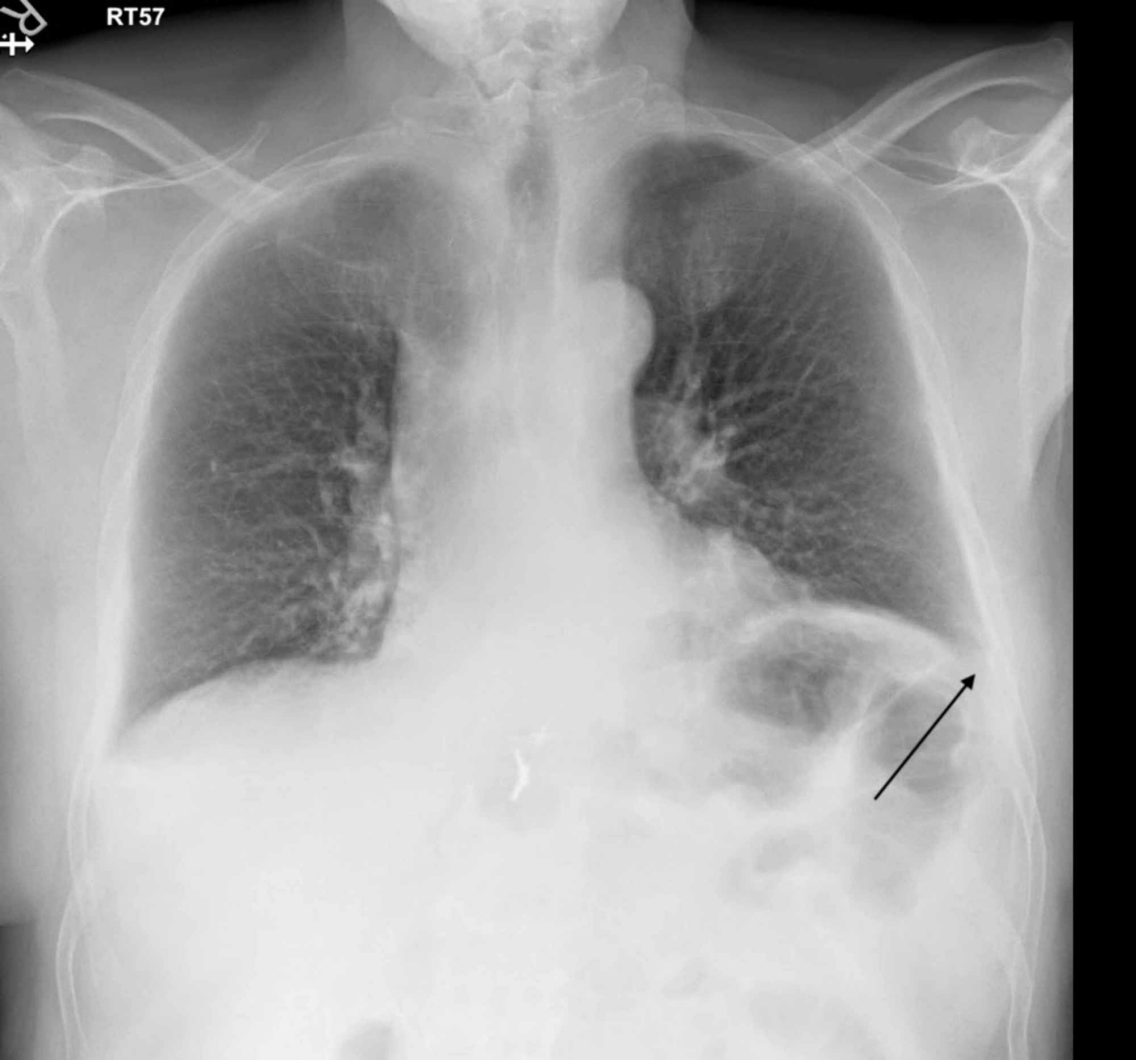
Right-sided pleural effusion with no mediastinal shift or pneumothorax

Laboratory investigations showed a white blood cell (WBC) count of 11.47 10^9/L, hemoglobin 154 g/L, platelet of 143 10^9/L. Liver function was within the patient’s baseline readings showing alanine transaminase of 97 U/L, aspartate aminotransferase 52.2 U/L, alkaline phosphatase 123 U/L, gamma-glutamyl transferase 209 IU/L, and D-dimer of 2.93 ug/ml fibrinogen equivalent units (FEU). Coagulation profile results when there is international normalized ratio (INR) of 1, prothrombin time of 12.8 seconds, and partial thromboplastin time 30.8 seconds. A chest X-ray showed right-sided pleural effusion with no mediastinal shift (Figure 1).

**Figure 2:**
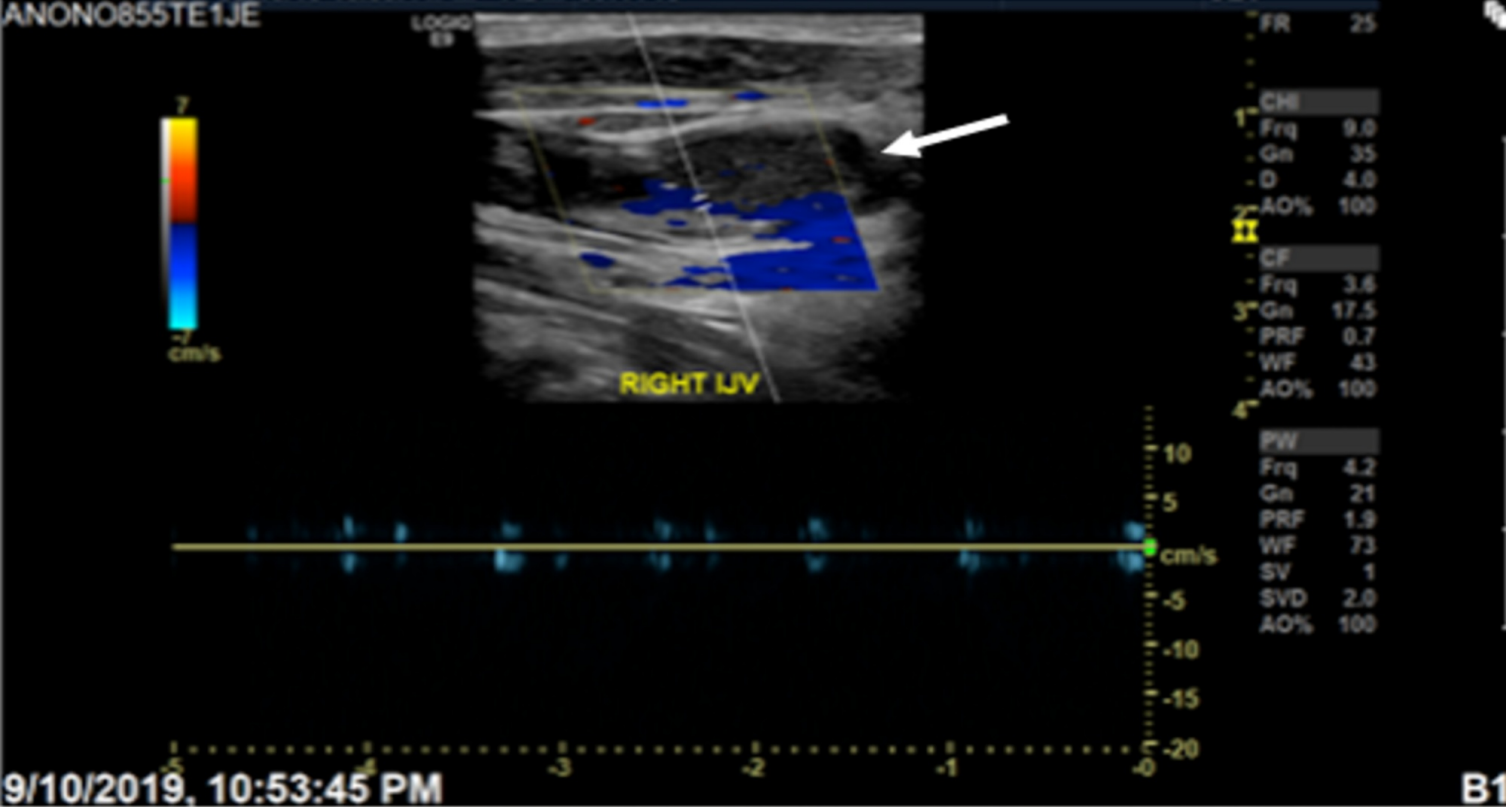
Filling defect observed in the right internal jugular vein

Upper extremity and neck ultrasound showed positive DVT of the right internal jugular vein, right subclavian vein, and axillary vein (Figure 2). The rest of the visualized veins showed good potency and flow.

**Figure 3:**
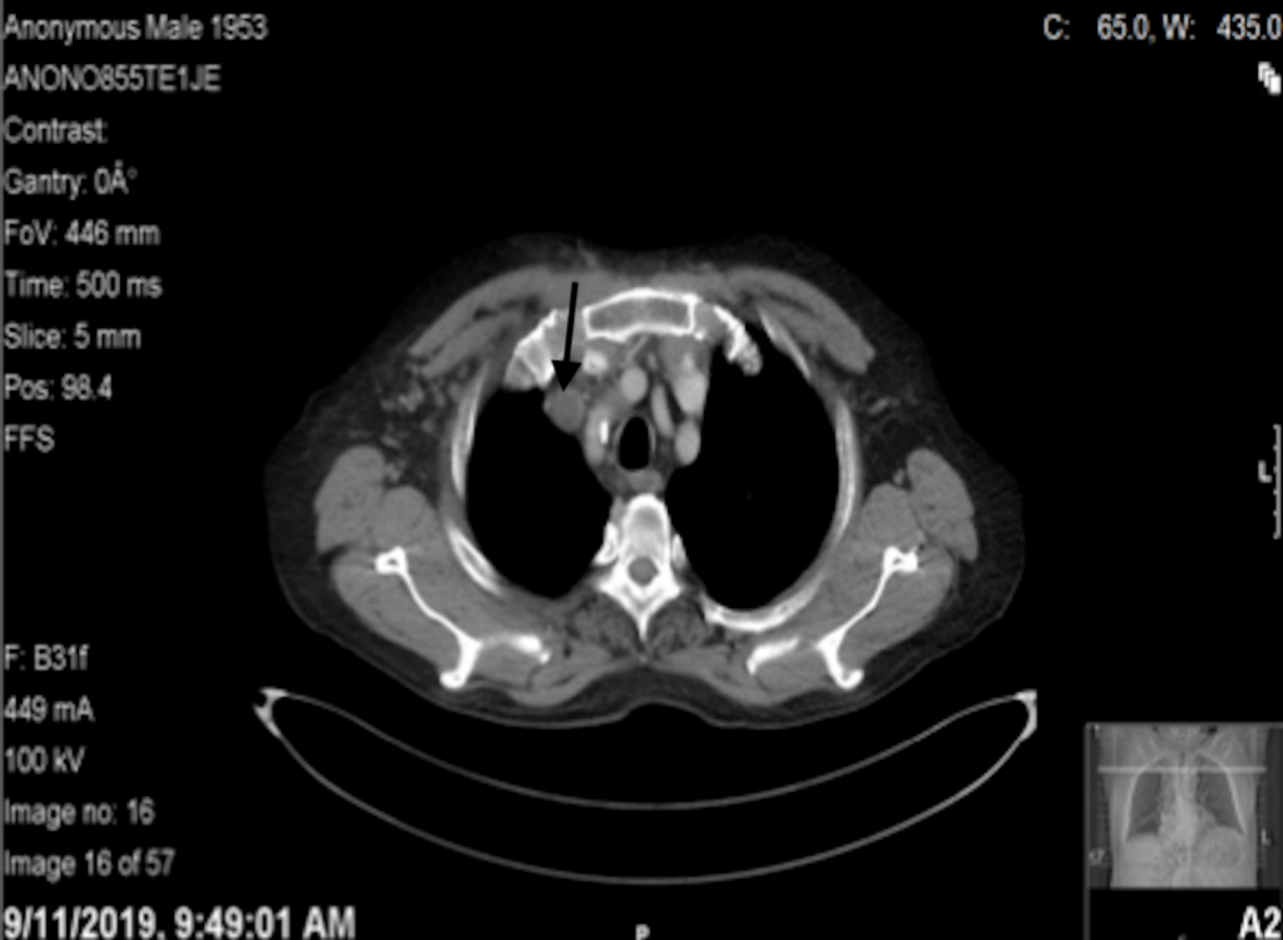
Hypodensity observed in the right internal jugular vein

**Figure 4:**
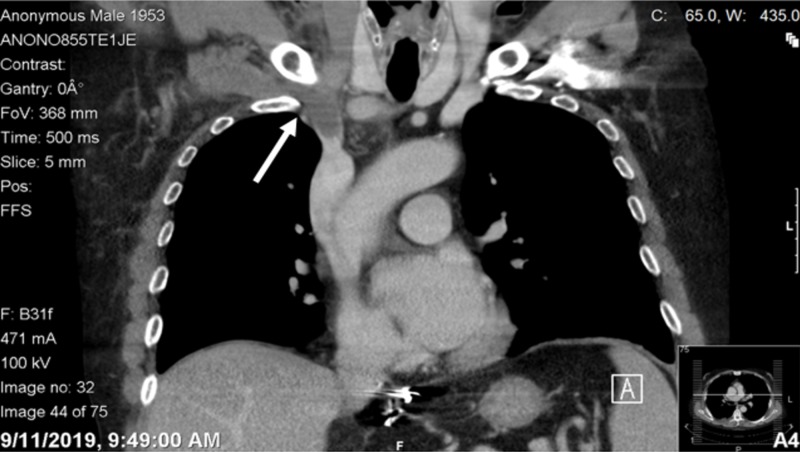
Coronal view of chest computed tomography showing hypodensity in the right internal jugular vein and in the subclavian veins

Computed tomography (CT) revealed thrombosed right internal jugular and subclavian veins; however, the superior vena cava is patent with no associated adjacent masses; small right-sided pleural effusion with basilar atelectasis was also noted (Figures 3-4).

General internal medicine service were consulted and after reviewing the patient’s case they prescribed Emxparine 1 mg/kg twice daily, along with close follow-ups at their clinic.

## Discussion

Internal jugular vein thrombosis (IJVT) is an elusive vascular disease that is rarely seen and results in potentially lethal complications [4]. UEDVT can be further classified into primary, spontaneous or secondary. DVT of the arm veins that happens without any apparent risk factors is classified as primary; those patients most likely have an underlying anatomic abnormality involving the thoracic outlet that caused the thrombus to form.

Secondary UEDVT accounts for up to 80% of UEDVT and is defined as any UEDVT associated with a predisposing factor, such as central venous catheters, implantable cardiac rhythm devices, malignancy, or insertion of other prosthetic or foreign material [5]. Other related risk factors include personal or family history of thrombosis and thrombophilia, surgery, trauma or immobilization of the arm, pregnancy and oral contraceptive use [6]. There have also been cases of UEDVT occurring in the dominant arm after strenuous, repetitive or unusual physical activity, such as lifting weights, playing tennis, pitching a baseball, or performing repetitive overhead activities such as painting.

Most patients are asymptomatic on presentation; however, some do present with the classic signs of DVT which include erythema, swelling, and tenderness [7]. Patients who present with septic thrombophlebitis, severe neck pain, or post head and neck trauma are prone to have Lemierre syndrome (necrobacillosis) [7]. On physical examination, swelling in the lateral neck from the angle of the mandible to the supraclavicular area over the anterior surface of the sternocleidomastoid muscle may be noted as well as superficial varicose collateral veins [7].

D-dimer is widely used as a testing modality for lower limb thrombosis, and has been heavily tested and found to be highly sensitive and non-specific for the thrombo-embolic events [8]. As for upper extremity thrombosis, D-dimer was found to have a negative predictive value of 93% according to Sartori et al. [9]; when it comes to confirming the diagnosis, an imaging modality is a must. The gold standard diagnostic modality for upper extremity DVT is contrast venography; however, it is not widely used due to being an invasive procedure associated with a number of complications [9]. Having a high clinical suspicion for upper extremity thrombosis with negative initial ultrasound results is an indicator for doing a venogram [10]. Thus, making the Duplex ultrasonography the first-line diagnostic imaging modality with 97% sensitivity and 96% specificity [11]. Nonetheless, when suspecting subclavian and brachiocephalic veins thrombosis, the anatomical relation to the clavicles overlying these vascular structures makes it difficult to visualize and compress the veins, limiting the accuracy of the study; hence considering CT venography is valid to complete the evaluation of the suspected thrombosis [12]. Magnetic resonance imaging is another alternative for visualizing subclavian and brachiocephalic veins thrombosis due to its abilities to clarify all the wanted vasculature [12]. 

## Conclusions

Upper limb thrombosis involves the deep venous structures of the arm and neck. There are two categories - primary and secondary venous thrombosis, the latter being provoked by an underlying pathology and the primary is unprovoked, meaning it is idiopathic. Primary upper limb venous thrombosis is considered an unusual case and is rare in incidence. In our case, we emphasized on the increased prevalence of primary internal jugular and subclavian veins thrombosis; based on that, we recommend physicians to have high clinical suspicion and proper diagnostic approach to detect upper limb thrombosis.

## References

[1] Lee Y, Siddiqui WJ (2019). Internal Jugular Vein Thrombosis. National Library of Medicine, 29 Apr.

[2] Louw VJ, Ntusi NA (2019). Virchow's triad revisited. S Afr Med J.

[3] Joffe HV, Goldhaber SZ (2002). Upper-extremity deep vein thrombosis. Circulation.

[4] Serinken M, Karcioglu O, Korkmaz A (2010). Spontaneous internal jugular vein thrombosis: a case report. Kaohsiung J Med Sci.

[5] Toratani M, Hayashi A, Nishiyama N (2017). Thrombosis in an internal jugular vein and an upper limb deep vein treated with edoxaban. Intern Med.

[6] Engelberger RP, Kucher N (2012). Management of deep vein thrombosis of the upper extremity. Circulation.

[7] Gbaguidi X, Janvresse A, Benichou J, Cailleux N, Levesque H, Marie I (2010). Internal jugular vein thrombosis: outcome and risk factors. QJM Int J Med.

[8] Goodacre S, Sampson FC, Sutton AJ, Mason S, Morris F (2005). Variation in the diagnostic performance of D-dimer for suspected deep vein thrombosis. QJM Int J Med.

[9] Sartori M, Migliaccio L, Favaretto E, Cini M, Legnani C, Palareti G, Cosmi B (2015). D-dimer for the diagnosis of upper extremity deep and superficial venous thrombosis. Thromb Res.

[10] Di Nisio M, Van Sluis GL, Bossuyt PM, Büller HR, Porreca E, Rutjes AW (2010). Accuracy of diagnostic tests for clinically suspected upper extremity deep vein thrombosis: a systematic review. J Thromb Haemost.

[11] Noyes AM, Dickey J (2017). The arm is not the leg: pathophysiology, diagnosis, and management of upper extremity deep vein thrombosis. RI Med J.

[12] Kraaijpoel N, van Es N, Porreca E, Büller HR, Di Nisio M (2017). The diagnostic management of upper extremity deep vein thrombosis: a review of the literature. Thromb Res.

